# Editorial: The Development of New Classes of Hypoxia Mimetic Agents for Clinical Use

**DOI:** 10.3389/fcell.2019.00120

**Published:** 2019-06-26

**Authors:** Ruoli Chen, Nicholas Forsyth

**Affiliations:** School of Pharmacy and Bioengineering, Keele University, Staffordshire, United Kingdom

**Keywords:** hypoxia inducible factor, inhibitor, 2 oxogluterate, hypoxia mimetic agents, prolyl hydroxylase (PHD)

Hypoxia-inducible factors (HIFs) are evolutionarily conserved transcription factors that regulate the bulk of hypoxic transcriptional responses in most mammalian cells. Transcriptionally active HIF levels rise at sub-physiological concentrations of oxygen inducing upregulation of a range of genes with activities ranging from cell protection to apoptosis according to the specific context and cell type. In humans, there are three known HIF isoforms; HIF-1 (first described in 1992) (Semenza and Wang, 1992) with a dominant role in the response to acute hypoxia, HIF-2 driving the response to chronic hypoxia, and HIF-3, a negative regulator of hypoxia-inducible gene expression (Graham and Presnell, 2017). Prolyl hydroxylase domain enzymes (PHDs), first described in 2001, provided the mechanism for HIF regulation via hydroxylation (Bruick, 2001). The PHDs themselves are 2-oxoglutarate (2OG) and non-heme-Fe(II)-dependent dioxygenase family members, all requiring Fe^2+^, 2OG, O_2_, and ascorbate for catalytic activity. Following on from their discovery three PHD isoforms (PHD1-3) have now been identified in mammalian tissue. PHD2, in particular, due to its slow reaction with oxygen, was identified as the key human oxygen sensor (Berra et al., 2003).

The direct connection between hydroxylase-dependent catalysis and physiological responses to hypoxia identify modulation of HIF hydroxylase activity as a potential therapeutic for use in a range of disease states including anemia, ischaemic diseases, and cancer. The term “hypoxia mimetic agent” is now widely used to indicate biological or chemical molecules which are used to activate the HIF pathway. Chemicals with a similar structure to 2OG, a tricarboxylic acid cycle (TCA) intermediate and a substrate of hydroxylase, compete with 2OG for binding sites at HIF prolyl hydroxylases and asparaginyl hydroxylase, factor inhibiting HIF (FIH), inhibit their activities, and stabilize HIF.

At current standing we are aware of four HIF hydroxylase inhibitors in clinical use or trial for anemia treatment in patients with chronic kidney disease (CKD). Small molecule HIF hydroxylase inhibitors have a number of advantages over other drugs such as low price, good compliance, and few allergic reactions. The pleiotropic and contrasting nature of the hypoxic response, driven by the large number of components involved, and the abundance of human 2OG oxygenases, ~60, create an importance around the development of highly potent and selective PHD inhibitors for clinical use (Yeh et al., 2017). This special issue is focused on the current development of hypoxia mimetic agents for clinical uses. We have covered a wide range of topics to highlight state of the art, limitations, and promises in the field, of interest to a broad community of researchers engaged in drug discovery.

In this special issue, Davis et al. have reviewed the application of PHD inhibitors for the treatment of ischaemic stroke. Neuroprotection via HIF-1α stabilization for ischaemic stroke is still a controversial topic; HIF downstream genes mediate both adaptive and pathological processes in stroke. Table 2, Davis et al., provides a valuable summary of studies with PHD inhibitors in preclinical stroke studies drawing the conclusion that the beneficial effects of HIF inhibition overweigh the side effects of the chemicals. The neuroprotection conferred by these agents unsurprisingly but importantly depends on the cell type and magnitude of ischaemic injury where some harmful effects of HIF activation can be eliminated by choosing the optimal window for PHD inhibitor administration.

Chen et al. emphasize that HIF PHD inhibition is not the same as HIF activation. Although HIF-α is the best studied substrate of PHD, the PHD has a number of other substrates (Chen et al., 2012). The inhibition of HIF hydroxylase not only exerts pleiotropic neuroprotective effects as a consequence of HIF induction, but also has anti-oxidant and anti-inflammatory effects. HIF has dual roles in reactive oxygen species (ROS) formation: reduction by suppression of mitochondrial TCA, or increase via NADPH oxidase, a HIF pathway target gene. Nevertheless, HIF PHD inhibition reduced ROS formation during ischemia/reperfusion. Developing further understanding of the HIF pathway and its relation with ROS may facilitate the advancement of pharmacological therapies for ischaemic stroke.

Wu et al. systemically review the role of hypoxia, HIF and PHD inhibitors in neural stem cell (NSC) proliferation and differentiation. The main NSC niches are located in the hippocampus and the subventricular zone at 1–5% O_2_. Mild hypoxia (2.5–5% O_2_) is the optimal condition for the proliferation of NSCs in comparison with 1 or 21% O_2_. Hypoxia promotes the proliferation of NSCs via a number of signaling pathways, such as the WNT/β-catenin, MEK/ERK and the PI3K/AKT signaling pathways. Hypoxia preconditioning increases NSC proliferation via the HIF-1α/AKT pathway. Additionally, WNT/β-catenin signaling is implicated in the effects of HIF-1α on NSC proliferation. Lastly, the authors described how the employment of hypoxia mimetic agents to provide HIF-1α stabilization might facilitate the expansion of NSCs *in vitro*. Novel compounds (e.g., FG4497) can induce neurogenesis without causing toxicity. Thus, the development of new specific and effective PHD inhibitors could offer a valid tool for the application of NSC transplantation in neurodegenerative disease.

Finally, Al Tameemi et al. review the effects of hypoxia on cancer cell metabolism. Hypoxia induces a number of protein changes in tumor cells which may initiate cell cycle arrest, differentiation, necrosis, and apoptosis, as well as stimulate tumor growth, invasion, and metastasis by facilitating acclimatization and survival. Under atmospheric oxygen conditions (21% O_2_), *in vitro* stem cells lose their stemness characteristics. *In vivo* normal adult stem cells are maintained in hypoxic conditions, typically defined as 1% oxygen. The stimulation of cancer cell stemness by HIFs is well-documented, with studies revealing HIF-1α and HIF-2α to be central to cancer stem cells stemness. The stemnesss stimulation is influenced more by HIF-2α (e.g., via Notch, Oct4, Sox2) than by HIF-1α (e.g., via glycolysis pathway). Furthermore, HIF-1α demonstrates tumor promoting capability. It is proposed that activating HIF-1α initiates autophagy and aerobic glycolysis; this provides cells surrounding the cancer cells with energy necessary to promote their growth. Glycolysis in tumors could be driven by HIF-1α stabilization, independently of the hypoxic environment. Additionally, HIF-1α enables cells to regulate to the reduced intracellular pH that occurs as a consequence of increased anaerobic glycolysis and the resulting lactic acid production.

In conclusion, the present issue has brought together, within a single place, some of the latest and most critical developments in current and emerging hypoxia mimetic agents. Roxadustat (FG4592) is currently licensed for treatment of CKD in China while other HIF PHD inhibitors are in phase II and III clinical trials. The HIF pathway modulates the expression of hundreds of human genes and for this reason more specific and potent small molecules to inhibit PHD are needed for the treatment of a wider range of diseases. Currently, no single drug specifically inhibits PHD isoforms and FIH, but some do provide differential inhibition. Agents stabilizing HIF via FIH inhibition warrant further exploration.

## Author Contributions

All authors listed have made a substantial, direct and intellectual contribution to the work, and approved it for publication.

### Conflict of Interest Statement

The authors declare that the research was conducted in the absence of any commercial or financial relationships that could be construed as a potential conflict of interest.
